# The Impact of Compulsory Citizenship Behavior on Organizational Silence in Nurses: The Roles of Presenteeism and Psychological Inflexibility

**DOI:** 10.1155/jonm/5341031

**Published:** 2026-03-15

**Authors:** Min Guo, Haixia Yang, Wenping Tang, Dandan Wang, Jingjing Ding, Xianwen Li

**Affiliations:** ^1^ School of Nursing, Nanjing Medical University, Nanjing, Jiangsu, China, njmu.edu.cn; ^2^ Nursing Department, Second Affiliated Hospital of Xi’an Jiaotong University, Xi’an, Shaanxi, China, xjtu.edu.cn; ^3^ Nursing Department, Kangda College of Nanjing Medical University, Lianyungang, Jiangsu, China, njmu.edu.cn; ^4^ Sir Run Run Hospital Nanjing Medical University, Nanjing, Jiangsu, China

**Keywords:** compulsory citizenship behavior, nurses, organizational silence, presenteeism, psychological inflexibility

## Abstract

**Background:**

Compulsory citizenship behavior (CCB) and organizational silence are prevalent among nurses, undermining nursing care quality and potentially threatening patient safety. Prior studies have shown that CCB can lead to employee silence; however, this relationship has not been examined among nurses, and the underlying mechanisms and boundary conditions remain unclear.

**Aim:**

This study aimed to test whether presenteeism mediates the association between CCB and organizational silence, with psychological inflexibility serving as a moderator of this process.

**Methods:**

A cross‐sectional study was conducted among nurses recruited via convenience sampling from three hospitals in Xi’an, Shaanxi Province, China. Four questionnaires were administered to assess CCB, presenteeism, organizational silence, and psychological inflexibility. A total of 400 valid responses were analyzed using Hayes’ PROCESS macro in SPSS to test a moderated mediation model.

**Results:**

Presenteeism mediated the relationship between CCB and organizational silence (*β* = 0.085, 95% CI: 0.039–0.131). Psychological inflexibility moderated the association between CCB and presenteeism: the positive effect of CCB on presenteeism was stronger among nurses high in psychological inflexibility (simple slope = 0.398, *p* < 0.001) than among those low in psychological inflexibility (simple slope = 0.217, *p* < 0.001).

**Conclusion:**

Presenteeism helps explain how CCB relates to organizational silence among Chinese nurses, and psychological inflexibility strengthens the adverse effect of CCB on presenteeism. Reductions in CCB and presenteeism may be associated with lower levels of organizational silence.

**Implications for Nursing Management:**

To improve nursing care and public health, healthcare managers should consider interventions aimed at reducing nurses’ experiences of CCB and presenteeism to help decrease organizational silence. In addition, strategies to reduce psychological inflexibility may help nurses better cope with demanding tasks and stressful work environments.

## 1. Introduction

The growing demand for high‐quality care—driven by shifts in disease patterns, population aging, and rising living standards—highlights the central role of nurses in healthcare quality. Understanding work stressors that shape nurses’ behavioral responses is therefore important for improving nursing practice and safeguarding patient care. Organizational citizenship behavior (OCB) refers to discretionary actions not formally rewarded yet beneficial to organizational functioning [1]. However, employees may sometimes perform such behaviors under external pressure. Vigoda‐Gadot [2] termed this pressure‐driven form compulsory citizenship behavior (CCB), and prior work has linked CCB to adverse job outcomes, including poorer performance and stronger turnover intention. In the Chinese context, relatively high power distance and collectivist norms may increase susceptibility to pressure to comply with extra‐role demands, potentially making CCB more prevalent [3].

Despite these concerns, the potentially detrimental consequences of CCB remain insufficiently understood, particularly regarding counterproductive work behavior [4]. Organizational silence refers to employees’ intentional withholding of opinions or information about organizational problems [5]. Evidence suggests that organizational silence is common among nurses and is associated with unfavorable outcomes such as lower professional identity and work engagement and higher turnover intention [6, 7]. Silence can also impede information exchange and error correction, thereby undermining decision‐making and patient safety [8]. Accordingly, organizational silence can be viewed as a passive form of counterproductive work behavior [9]. Because CCB represents an externally imposed, resource‐draining demand, we hypothesized that CCB would be positively associated with organizational silence among nurses.

To better elucidate this relationship, our second aim was to clarify the mechanism through which CCB may contribute to organizational silence. Conservation of resources theory (COR) suggests that individuals strive to acquire and protect valued resources and that stress emerges when these resources are threatened or depleted [10]. CCB can be viewed as a resource‐draining work demand that may erode employees’ energy and psychological resources, thereby increasing reliance on coping behaviors aimed at conserving remaining resources. Presenteeism—being at work while not fully functioning [11]—is common in nursing and often involves attending work despite illness and/or reduced performance due to stress [12]. Nurses report higher levels of presenteeism than many other occupational groups [13, 14]. In the Chinese work context, attending work while unwell may at times be interpreted as diligence and commitment [15], which could make presenteeism a salient, resource‐conserving response when employees experience heightened demands. Nurses facing CCB‐related resource depletion may engage in presenteeism to preserve role performance and interpersonal standing, while simultaneously limiting discretionary, potentially risky behaviors such as speaking up. Although research has seldom examined the association between presenteeism and organizational silence, the link is conceptually plausible. Working while ill is often accompanied by fatigue and reduced motivation [16], which may diminish employees’ capacity and willingness to voice concerns and increase the tendency to withhold input. Together, these arguments suggest that CCB may relate to organizational silence both directly and indirectly through increased presenteeism. Accordingly, we hypothesized that CCB directly and indirectly predicts organizational silence via the mediating role of presenteeism.

Because daily stress is omnipresent, identifying actionable internal resources is critical when designing interventions to reduce stress‐related harm. Considering the highly demanding nature of nurses’ work environments, promoting factors that protect nurses’ physical and mental health is especially important. Personal resources can buffer the detrimental consequences of stressful events, and psychological flexibility has attracted increasing attention as an important such resource. Within the acceptance and commitment therapy framework, psychological inflexibility reflects a rigid coping style marked by experiential avoidance and cognitive fusion, which can intensify distress and constrain adaptive responding under stress [17, 18]. Prior evidence indicates that psychological flexibility buffers the impact of stressors on mental and physical health [19]. Accordingly, we propose that psychological inflexibility may moderate the relationship between CCB (stressors) and presenteeism (negative outcomes).

Presenteeism leads to ongoing depletion of key resources. In such a state, speaking up or voicing suggestions requires additional cognitive and emotional investment and may entail interpersonal conflict or extra workload; consequently, voice is more likely to be appraised as a high‐cost and high‐risk behavior, increasing the likelihood of organizational silence [5]. Individuals high in psychological inflexibility tend to have difficulty in flexibly regulating discomfort and negative emotions and are more sensitive to and persistently preoccupied with perceived resource loss [17]. As a result, when resources are depleted due to presenteeism, psychologically inflexible employees may be more inclined to interpret voice as threatening and to rely on avoidance‐based coping, thereby strengthening the link between presenteeism and organizational silence [20]. Moreover, as an involuntary and externally imposed demand, CCB may directly trigger resource depletion and perceptions of unfairness, which can activate avoidance‐oriented, self‐protective motivation [21]. Psychological inflexibility may further amplify this process: highly inflexible individuals are more prone to ruminate on negative interpretations and become emotionally entangled and are less able to employ flexible cognitive reappraisal or values‐based action to buffer stress. Consequently, they may evaluate speaking up as ineffective or unsafe, thereby intensifying the direct positive association between CCB and organizational silence. Although theorizing specifically regarding moderation on these downstream paths remains limited, some preliminary indications in related research suggest that psychological inflexibility may also shape both the presenteeism–organizational silence relationship (Path b) and the direct association between CCB and organizational silence (Path c’). Therefore, we exploratory test whether psychological inflexibility moderates the relationship between presenteeism and organizational silence, as well as the direct association between CCB and organizational silence.

## 2. Methods

### 2.1. Design and Participants

A cross‐sectional survey was conducted from January to March 2024. The STROBE checklist was adhered to in this study. The study was conducted in three tertiary hospitals selected by convenience sampling in Xi’an, China. The inclusion criteria were as follows: (1) certified registered nurses, (2) full‐time workers, and (3) volunteering to participate in the study. Nurses not on duty during the survey period were excluded. The sample size was determined using G^∗^Power; considering 13 predictors, including independent variables, mediators, moderators, and sociodemographic characteristics, a medium effect size (*f* ^2^ = 0.15), a power of 0.80, and an 0.05 alpha level [22], a sample size of at least 131 participants was necessary. The final sample size of 400 participants complies with the requirement.

### 2.2. Measures

#### 2.2.1. Sociodemographic Characteristics

The sociodemographic variables of interest in this study were age, gender, education level, years of nursing experience, night shifts, professional title, department, marital status, number of children, and monthly income.

#### 2.2.2. CCB

The CCB was measured using a single‐dimensional, five‐item, self‐reported instrument [2], with a sample item being “Under pressure from the organization, I need to make extra efforts to meet job requirements.” Each item was answered on a five‐point Likert scale, with total scores ranging from 5 to 25. This scale has been tested in Chinese organizational contexts and has shown good reliability and validity [23]. The scale had a Cronbach’s alpha of 0.902 in this study.

#### 2.2.3. Presenteeism

The six‐item, self‐reported Stanford Presenteeism Scale was used to measure presenteeism [24]. Items were answered on a five‐point Likert scale, with total scores ranging from 6 to 30 and being calculated by adding the scores for the six items. Higher ratings suggest more significant health‐related productivity loss. This scale has been tested in a Chinese context, showing acceptable psychometric properties [25]. In this study, the scale had a Cronbach’s alpha value of 0.889.

#### 2.2.4. Organizational Silence

Organizational silence was measured using the 20‐item organizational silence scale [26]. Items were answered on a five‐point Likert scale, with total scores ranging from 20 to 100 and being calculated by adding the scores for the 20 items. Higher scores implied a higher level of organizational silence. Acceptable psychometric properties have been demonstrated by testing the scale in a Chinese setting [8]. The scale’s Cronbach’s alpha in this investigation was 0.969.

#### 2.2.5. Psychological Inflexibility

Psychological inflexibility was assessed using the 30‐item psychological inflexibility subscale of the Multidimensional Psychological Flexibility Inventory [27]. Items were answered on a six‐point scale, with total scores ranging from 30 to 180 and being calculated by adding the 30 items. Higher scores indicated greater levels of psychological inflexibility. The study that translated the scale into Chinese found that it had acceptable psychometric properties in the Chinese setting [28]. The scale’s Cronbach’s alpha in this investigation was 0.941.

### 2.3. Ethical Considerations

This investigation was approved by the ethics committee of XXX (No: XXX) [details blinded for review]. Every participant provided informed consent.

### 2.4. Data Collection

Online questionnaires (https://www.wjx.cn) were used for anonymous data collection. Before the formal survey, a pilot survey was carried out with 10 nurses to assess item clarity and to identify potential technical problems in using the online questionnaire. The initial page of the questionnaire introduced the study’s aim and content and provided information about confidentiality, anonymity, and voluntary participation. Three investigators were trained to manage the online platform and monitor questionnaire quality. After obtaining approval from hospital administrators, the same survey link and standardized recruitment instructions were sent to the nursing managers in each of the three hospitals. Nursing managers assisted in disseminating the survey link to eligible nurses and delivered identical instructions across sites. Data collection was conducted concurrently across all three hospitals during the same overall period (January to March 2024). No incentives were provided, and no changes were made to the questionnaire or procedures during the data‐collection period.

### 2.5. Data Analysis

Data were analyzed using SPSS version 29.0. The Harman single‐factor test was used to test common method bias. Correlations between the study variables (CCB, presenteeism, organizational silence, and psychological inflexibility) were tested using Pearson’s correlation. The moderated mediation model was analyzed using the PROCESS macro in SPSS [29]. Model 4 was used to test the mediating effect of presenteeism on the relationship between CCB and organizational silence. Model 59 was used to examine the moderated mediation effect. To further understand the moderating effect of psychological inflexibility, a simple slope analysis was conducted. Sociodemographic variables were controlled for as covariates when the mediating and moderating effects were analyzed. The *p*‐values were two‐tailed, and a *p* < 0.05 was considered statistically significant. Consistent with recent empirical research employing regression‐based conditional process and moderation analyses to examine boundary conditions in organizational contexts [30], Hayes’ PROCESS macro provides an appropriate analytic framework for testing mediation, moderation, and moderated mediation effects.

## 3. Results

### 3.1. Participants’ Characteristics

Following contemporary recommendations for improving data quality in online surveys, we conducted a systematic screening for careless responding prior to hypothesis testing. Four indices were computed across all 61 items: longstring response (LS), person‐total variance (PTV), Mahalanobis distance squared (MD^2^), and response time (RT). Decision thresholds were defined a priori using percentile‐based cutoffs calculated on the full raw sample (*N* = 435): RT in the bottom 10% (RT ≤ 294.4 s), LS ≥ 31, PTV in the bottom 10% (PTV ≤ 0.43), and MD^2^ in the top 10% (MD^2^ ≥ 114.26). To reduce false positives associated with any single indicator, respondents were excluded only if they met at least two of the four criteria (≥ 2). This procedure identified 35 potential careless respondents, yielding a final cleaned dataset of *N* = 400 for all subsequent analyses.

Participants’ mean age was 33.73 (±7.58) years (range 21–57 years), and the mean years of nursing experience were 11.16 years. Most participants were women (97.5%, *n* = 390). Furthermore, 91.3% (*n* = 365) of participants had a bachelor’s degree. Most participants were married (70.5%, *n* = 282) and had an income of 5000–10,000 yuan per month (70.5%, *n* = 282). The professional titles consisted of 68 juniors (17.0%), 164 seniors (41.0%), 148 nurses in charge (37.0%), and 20 associate professors and professors (5.0%). The participants worked in the following departments: internal medicine (23.8%, *n* = 95), surgery (28.3%, *n* = 113), obstetrics and gynecology (6.5%, *n* = 26), pediatrics (16.0%, *n* = 64), and other departments (25.5%, *n* = 102). More details about participants’ characteristics are presented in Table 1.

**TABLE 1:** Descriptive statistics of participants’ characteristics.

Variable	Mean ± SD/*n* (%)
Age (years)	33.73 ± 7.58
Gender	
Male	10 (2.5%)
Female	390 (97.5%)
Education level	
Junior college education	19 (4.8%)
Bachelor’s degree	365 (91.3%)
Master’s degree or above	16 (4.0%)
Years of nursing experience	11.16 ± 8.35
Monthly average frequency of night shifts	7.17 ± 5.05
Professional title	
Junior registered nurses	68 (17.0%)
Senior registered nurses	164 (41.0%)
Nurses in charge	148 (37.0%)
Associate professor and professor nurses	20 (5.0%)
Department	
Internal medicine	95 (23.8%)
Surgery	113 (28.2%)
Obstetrics and gynecology	26 (6.5%)
Pediatrics	64 (16.0%)
Others	102 (25.5%)
Marital status	
Single	115 (28.7%)
Married	282 (70.5%)
Divorced or separated	3 (0.8%)
Number of children	
0	148 (37.0%)
1	173 (43.3%)
> 2	79 (19.8%)
Monthly income	
< 5000 yuan (US$700)	33 (8.3%)
5000–10,000 yuan (US$700–1400)	282 (70.5%)
> 10,000 yuan (US$1400)	85 (21.2%)

### 3.2. Descriptive Statistics and Correlation Analysis of Study Variables

As presented in Table 2, CCB correlated positively with presenteeism (*r* = 0.497, *p* < 0.001), organizational silence (*r* = 0.633, *p* < 0.001), and psychological inflexibility (*r* = 0.430, *p* < 0.001); presenteeism correlated positively with organizational silence (*r* = 0.437, *p* < 0.001) and psychological inflexibility (*r* = 0.386, *p* < 0.001); and organizational silence correlated positively with psychological inflexibility (*r* = 0.425, *p* < 0.001).

**TABLE 2:** Descriptive statistics and correlations of study variables.

Variables	Mean scores (mean ± SD)	1	2	3	4
1. CCB	12.95 (5.181)	1			
2. Presenteeism	15.42 (4.005)	0.497^∗∗∗^	1		
3. Organizational silence	48.23 (18.264)	0.633^∗∗∗^	0.437^∗∗∗^	1	
4. Psychological inflexibility	95.82 (24.285)	0.430^∗∗∗^	0.386^∗∗∗^	0.425^∗∗∗^	1

^∗∗∗^ *p* < 0.001.

### 3.3. Mediation Analysis

The mediating effect of presenteeism on the relationship between CCB and organizational silence was tested using Model 4. The results are summarized in Table 3. In Model 1, the effect of CCB on organizational silence was significant (*β* = 0.656, *p* < 0.001). In Model 2, the effect of CCB on presenteeism was significant (*β* = 0.497, *p* < 0.001). When the mediating variable was entered (Model 3), the direct effect of CCB on organizational silence remained significant (*β* = 0.572, *p* < 0.001), and the effect of presenteeism on organizational silence was also significant (*β* = 0.171 *p* < 0.001). Additionally, the 95% CI for the indirect effect of CCB on organizational silence through presenteeism did not contain zero (*β* = 0.085, 95% CI: 0.039–0.131), suggesting that presenteeism mediates the relationship between CCB and organizational silence.

**TABLE 3:** The mediating effect of presenteeism on the relationship between CCB and organizational silence.

	Organizational silence			Presenteeism			Organizational silence		
	*β*	*t*	*p*	*β*	*t*	*p*	*β*	*t*	*p*
CCB	0.656	16.311	< 0.001	0.497	10.955	< 0.001	0.572	12.621	< 0.001
Presenteeism							0.171	3.840	< 0.001
*R* ^2^	0.420			0.268			0.441		
*F*	25.534^∗∗∗^			12.938^∗∗∗^			25.465^∗∗∗^		

^∗∗∗^ *p* < 0.001.

### 3.4. Moderated Mediation Analysis

The results (Table 4) revealed that the interaction of CCB and psychological inflexibility had a significant effect on presenteeism (*B* = 0.004, *p* = 0.002), suggesting the moderation of psychological inflexibility of the relationship between CCB and presenteeism. The interaction of CCB and psychological inflexibility and the interaction of presenteeism and psychological inflexibility had no significant effect on organizational silence (*B* = −0.006, *p* = 0.346; *B* = −0.012, *p* = 0.209). The above results support that psychological inflexibility moderates only the first half of the indirect effect of the mediation model and hence not the second half of the indirect or direct effect.

**TABLE 4:** Moderated mediation model analysis.

	Presenteeism			Organizational silence		
	*B*	*t*	*p*	*B*	*t*	*p*
CCB	0.308	8.177	< 0.001	1.844	11.134	< 0.001
Psychological inflexibility	0.036	4.574	< 0.001	0.121	3.687	< 0.001
CCB × psychological inflexibility	0.004	3.116	0.002	−0.006	−0.943	0.346
Presenteeism				0.664	3.231	0.001
Presenteeism × psychological inflexibility				−0.012	−1.258	0.209
*R* ^2^	0.318			0.470		
*F*	13.825^∗∗∗^			22.698^∗∗∗^		

^∗∗∗^ *p* < 0.001.

**FIGURE 1:**
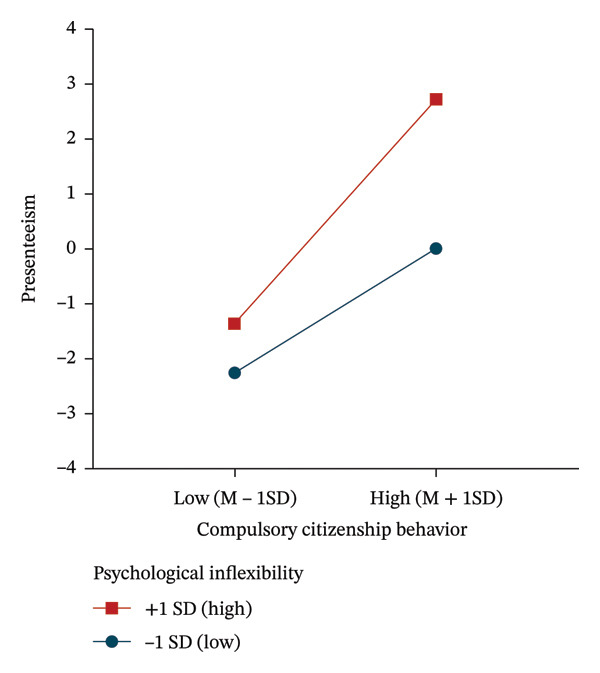
The moderating effect of psychological inflexibility on the relationship between CCB and presenteeism.

Simple slope analysis showed (Figure 1) that for participants with higher levels of psychological inflexibility (M + 1SD), CCB had a significant positive effect on presenteeism (simple slope = 0.398, *p* < 0.001), and this positive effect remained significant, albeit smaller, among participants with lower levels (M‐1SD; simple slope = 0.217, *p* < 0.001). These results suggest that as the level of psychological inflexibility increases, the positive effect of CCB on presenteeism increases.

## 4. Discussion

This study investigated the relationships between CCB, presenteeism, organizational silence, and psychological inflexibility in nurses using a moderated mediation model. The findings indicated that CCB, presenteeism, and psychological inflexibility correlated significantly with organizational silence; presenteeism mediated the association between CCB and organizational silence; the indirect effect of the mediation model was moderated by psychological inflexibility.

The mean organizational silence score among nurses in our sample (48.78) suggests a moderate level of silence and is comparable to prior nursing studies [8, 31]. By contrast, lower silence levels have been reported among healthcare workers in Switzerland [32], potentially because that sample included physicians, who tend to report less silence than nurses [33]. Cross‐national differences in organizational context and cultural norms (e.g., power distance) may also partly account for inconsistencies across settings. Given accumulating evidence that organizational silence undermines professional identity, engagement, and other work outcomes [6, 7], our findings highlight the need to monitor and address organizational silence among Chinese nurses. Regarding CCB, the mean total score (13.01) is similar to that reported among nurses in Turkey [34]. Notably, empirical evidence on CCB among Chinese nurses remains scarce, and future studies are needed to establish broader benchmarks. Presenteeism in our sample was also notable (total mean = 15.53), aligning with recent studies among Chinese nurses [35]. International evidence likewise indicates that presenteeism is prevalent in nursing, with substantial variation across contexts [36]. Taken together, these descriptive results suggest that CCB, presenteeism, and organizational silence are all salient phenomena in nursing and warrant continued attention in both research and practice.

Our finding is consistent with prior work linking CCB to employee silence, indicating that coerced extra‐role demands may be accompanied by greater tendencies to withhold voice and concerns [37]. More importantly, we found that presenteeism was positively associated with organizational silence and mediated the relationship between CCB and organizational silence. From a COR perspective, individuals strive to acquire, retain, and protect valued resources (e.g., energy, time, cognitive capacity, and emotional stability) [10]. Presenteeism, being physically present at work while functionally impaired, can be conceptualized as a state of substantial resource depletion, in which nurses must continue performing demanding tasks while coping with illness‐related fatigue, pain, and reduced concentration [38, 39]. Under such conditions, core personal resources (physical energy, attentional control, emotional regulation, and self‐efficacy) are continuously consumed simply to meet basic job requirements. Importantly, COR theory proposes a “desperation principle:” when individuals perceive that their remaining resources are nearing exhaustion, they may enter a defensive mode in which the primary goal shifts from gaining new resources to protecting the scarce resources that remain [10]. In this state, people prioritize behaviors that minimize additional losses and avoid situations that could trigger further depletion. This principle offers a clear psychological explanation for why presenteeism may translate into organizational silence among nurses. Speaking up in organizations is not a cost‐free act [5, 40]. Voicing concerns or suggestions typically requires additional cognitive and emotional investment (e.g., identifying problems, constructing coherent arguments, selecting an appropriate time and channel, and anticipating and managing possible disagreement or interpersonal tension). For nurses already operating under depleted resources, these additional demands may be appraised as both high‐cost and high‐risk. Therefore, organizational silence can be understood as a rational, defensive resource conservation strategy—an “energy‐saving” tactic aimed at preventing further resource loss [40]. Remaining silent allows exhausted nurses to reduce additional cognitive load and emotional labor and to avoid potential secondary losses (e.g., strained relationships, conflict with supervisors, or reputational risk). This mechanism may be further reinforced in the Chinese nursing context described in our study, where interpersonal harmony and relationship maintenance are culturally valued, making restraint and conflict avoidance more likely to be seen as adaptive strategies. Under presenteeism, when resources are scarce and self‐protection becomes central, choosing silence may simultaneously serve resource‐preserving and relationship‐preserving functions, thereby increasing the tendency to withhold voice.

In the moderated mediation model, psychological inflexibility significantly strengthened the association between CCB and presenteeism, whereas its interactions with presenteeism and CCB in predicting organizational silence were not significant. This pattern suggests that the moderating role of psychological inflexibility may be stage‐specific, operating primarily at the stressor‐to‐strain stage rather than at subsequent strain‐to‐behavior stages. Psychological inflexibility reflects a rigid coping style characterized by experiential avoidance and cognitive fusion, which tends to intensify how individuals appraise and internalize aversive work demands [41, 42]. When nurses encounter CCB as an interpersonal and organizational stressor, those higher in psychological inflexibility may experience greater emotional entanglement and difficulty disengaging from distressing thoughts and feelings, thereby accelerating resource depletion and increasing the likelihood of impaired functioning at work, such as presenteeism [13, 43]. This finding is consistent with broader evidence showing that psychological inflexibility amplifies the impact of stressors on strain‐related outcomes, including physical and mental health problems [44].

By contrast, once presenteeism has occurred, downstream behavioral responses such as organizational silence may be shaped more strongly by situational, relational, and normative constraints than by individual differences in psychological inflexibility. In the Chinese nursing context, organizational silence is often embedded in norms emphasizing compliance, deference to authority, and relationship maintenance [45, 46]. Under such conditions, remaining silent in response to CCB may represent a broadly shared, socially reinforced coping strategy, which could limit the extent to which psychological inflexibility further differentiates employees’ silence responses. Accordingly, the nonsignificant moderation effects on the presenteeism–silence association and the direct CCB–silence association should be interpreted as indicating that psychological inflexibility primarily conditions strain formation, rather than all subsequent behavioral manifestations of strain. Importantly, these moderation tests were exploratory in nature and warrant further investigation in future research using longitudinal or multilevel designs.

## 5. Implications

The present findings offer several actionable implications for nursing management and intervention design. First, because CCB was positively associated with both presenteeism and organizational silence, reducing CCB should be treated as a primary prevention target. Hospital administrators and nursing managers can intervene at the unit level by establishing clear anticoercion policies and implementing leadership training that emphasizes respectful supervision and fair workload allocation. Second, improving nurses’ health‐related functioning at work may help decrease silence even when CCB cannot be fully eliminated. Practical interventions include ensuring adequate staffing, enabling flexible scheduling, encouraging appropriate sick leave use, and providing recovery opportunities. Third, psychological inflexibility intensified the impact of CCB on presenteeism, suggesting that individual‐focused interventions may be especially beneficial for nurses who show higher inflexibility. Brief, scalable programs grounded in acceptance and commitment therapy principles—such as training in psychological flexibility, mindfulness‐based skills, and values‐consistent coping—may weaken the stressor‐to‐strain pathway.

### 5.1. Limitations

This study has several limitations. First, participants were recruited only from three hospitals in Xi’an, Shaanxi province, which may limit the generalizability of the findings. Future studies using more diverse samples across regions and healthcare settings are needed to replicate and extend the results. Second, although participants were drawn from multiple hospitals, our primary analyses did not explicitly model the nested structure (nurses nested within hospitals). To assess the magnitude of clustering, we computed intraclass correlation coefficients (ICC) for organizational silence and presenteeism using a one‐way random‐effects model. The ICC (1) values were 0.0127 (organizational silence) and 0.0261 (presenteeism), and the corresponding ICC (2) values were 0.6318 and 0.7814, respectively, indicating modest between‐hospital variance. However, because the number of hospitals was small (three), future research should include a larger number of hospitals and apply multilevel modeling to account for hierarchical data structures more rigorously. Third, the cross‐sectional design precludes causal inference; future longitudinal or multiwave designs are needed to examine temporal ordering among CCB, presenteeism, organizational silence, and psychological inflexibility.

## 6. Conclusion

This study is among the first to examine the interrelationships among CCB, presenteeism, organizational silence, and psychological inflexibility in a nursing context. Our findings indicate that higher levels of CCB are associated with greater presenteeism, which in turn is linked to increase organizational silence among Chinese nurses. Psychological inflexibility further intensifies the adverse association between CCB and presenteeism. Together, these results suggest that efforts to reduce nurses’ experiences of CCB and presenteeism may contribute to mitigating organizational silence. From a practical perspective, healthcare managers and policymakers may consider interventions aimed at limiting compulsory extra‐role demands and addressing presenteeism, alongside initiatives that foster psychological flexibility, to help nurses better cope with the high demands of their work environment and to support the quality of nursing practice and public health outcomes.

## Funding

The researchers affirm that they received no funding for conducting this study.

## Conflicts of Interest

The authors declare no conflicts of interest.

## Data Availability

The data that support the findings of this study are available from the corresponding author upon reasonable request.
